# Traitement des fractures de la diaphyse humérale par l’embrochage centro-médullaire rétrograde de Hackethal: à propos de 54 cas

**DOI:** 10.11604/pamj.2018.30.38.14589

**Published:** 2018-05-17

**Authors:** Ouahidi Mohamed, Hicham Bousbaa, Mourad Bennani, Toufik Cherrad, Hassan Zejjari, Jamal Louste, Khalid Rachid, Laarbi Amhajji

**Affiliations:** 1 Orthopedic Surgery and Traumatology, Military Hospital Moulay Ismail, Meknes, Morocco

**Keywords:** Fracture, diaphyse, humérus, hackethal, nerf radial, embrochage, Fracture, diaphysis, humerus, Hackethal, radial nerve, bundle nailing

## Abstract

Les auteurs rapportent 54 cas de fracture de la diaphyse humérale traités par embrochage de Hackethal. 3 cas avec paralysie du nerf radial ont été observé dont l'abstention thérapeutique en urgence a été l'attitude avec surveillance et qui ont abouti à une récupération. La technique de l'embrochage est relativement facile mais nécessite une technique rigoureuse et une bonne expérience de l'opérateur. Un taux de 7,84 % de pseudarthrose était observé. L'embrochage d'Hackethal peut être considérer comme un traitement orthopédique amélioré avec un coût modeste et une innocuité importante.

## Abstract (en)

We here report 54 cases of humerus diaphyseal fractures treated via Hackethal’s bundle nailing. We observed 3 cases of radial nerve palsy. Therapeutic abstention associated with monitoring was the applied attitude toward these patients on presentation to the Emergency Department. It resulted in recovery. Hackethal’s bundle nailing technique is relatively easy but it requires rigorous surgical procedure performed by an experienced surgeon. A rate of 7.84% of pseudarthrosis is reported. Hackethal’s bundle nailing is a very safe, improved orthopedic treatment with modest cost.

## Introduction

Les fractures de la diaphyse humérale représentent 5 % de toutes les fractures [1]. Le but de notre étude est de mettre le point sur la place de l´embrochage d´Hackethal et son intérêt dans les fractures de l'humérus qui sont de plus en plus fréquente au Maroc avec la recrudescence des accidents de la voie publique. Le diagnostic des fractures de l´humérus est facile et leurs complications les plus fréquentes sont la pseudarthrose et la paralysie du nerf radial. L'embrochage fasciculé d'Hackethal fait partie de nombreux moyens d'ostéosynthèses proposés.

## Méthodes

**Figure 1:**
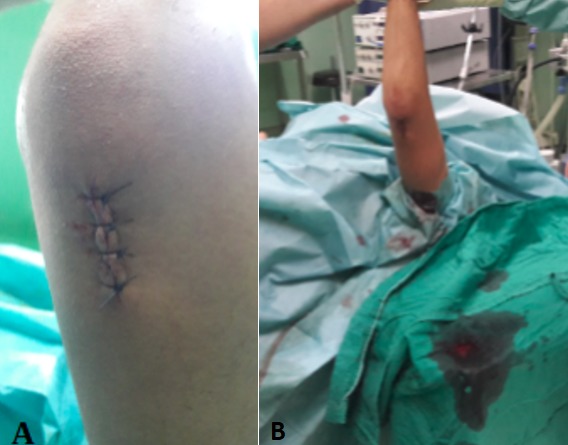
A) voie d’abord sus-olécranien; B) position en décubitus dorsal et membre supérieur sans garrot sur table radio-transparente

**Figure 2:**
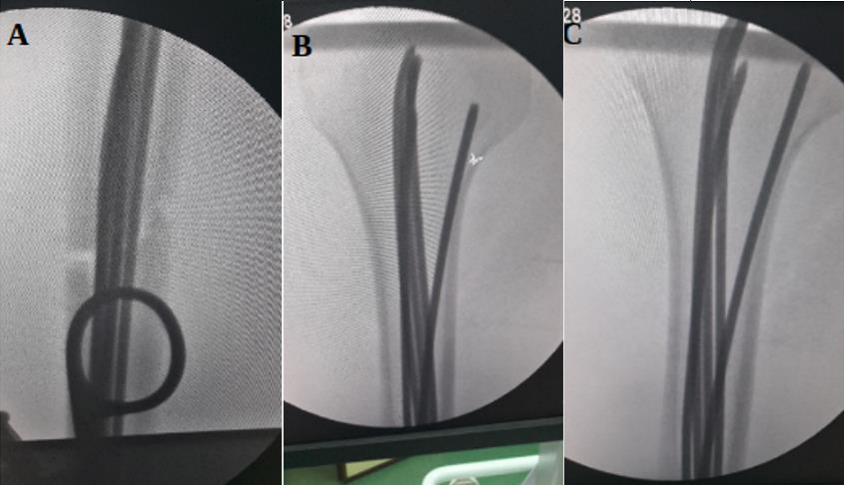
A) trait de fracture transversal réduit avec comblement du canal médullaire; (B, C) introduction de par 5 broches 30/10 jusqu’à la reégion médullaire de l’extrémité supérieur de l’humérus

**Figure 3:**
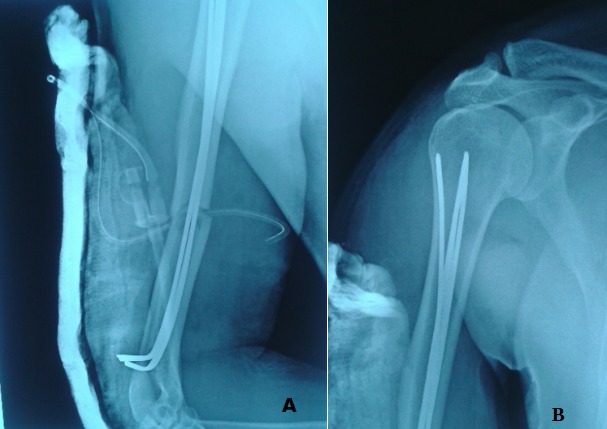
(A, B) fracture ouverte de l’humérus Cauchoix 2 opéré avec mise en place de 3 broches introduite au-dessus de la fossette sus-olécranien

Une série de 54 ostéosynthèses par embrochage ascendant de l´humérus a été revue chaque fois que le diamètre du canal médullaire le permet, à savoir un diamètre > 6mm et quel que soit le type de la fracture, durant une période de 6 ans entre juillet 2011 et Juin 2016. 3 patients ont été exclus de cette étude, car ayant été perdus de vue. Les résultats ont été évalués chez 51 patients (94,5%). Des variables d'ordre épidémiologique, clinique, paraclinique, thérapeutique et évolutif ont été analysées en se basant sur une fiche d'exploitation après convocation des malades. L'anesthésie générale a été effectuée dans 34 cas et le bloc plexique dans 17 cas. L´opération nécessite 2 aides. L´installation était faite en décubitus dorsal sur table ordinaire avec membre fracturé sans garrot sur table radio-transparente permettant l´utilisation d´un amplificateur de brillance, l´abord était presque toujours sus-olécranien (Figure 1). La trépanation était faite à l´aide d´une mèche permettant de réaliser un trou ovale puis un maximum de broches introduite dans le canal médullaire (Tableau 1) de taille entre 25/10 et 30/10 à extrémitées mousse et béquillées d'un angle d'environ 20° (Figure 2, Figure 3). Le montage était ensuite complété par une immobilisation type Dujarier pour 30 jours. Tous les malades ayant une fracture isolée de l´humérus ont quitté l'hôpital le lendemain. La rééducation passive est débutée en fonction de l'indolence sous couvert d´un bandage. Les patients étaient revus tous les mois jusqu'à 6 mois post-opératoire, puis tous les 3 mois. Le résultat fonctionnel était enregistré au moins 6 mois après l'opération. Nous avons utilisé la classification de Stewart et Hundley modifié pour étudier le résultat fonctionnel [**2**]. Le recul moyen était de 45 mois avec des extrêmes de 6 mois et 96 mois.

**Tableau 1:** Nombre de broches introduites et de pseudarthroses

Nombre de broches (n)	Nombre de patients (n)	Nombre pseudarthrose (n) (%)
1	1	1 (100)
2	3	1 (33,33)
3	37	1 (2,7)
4	6	0 (0)
5	4	1 (25)

## Résultats

**Figure 4:**
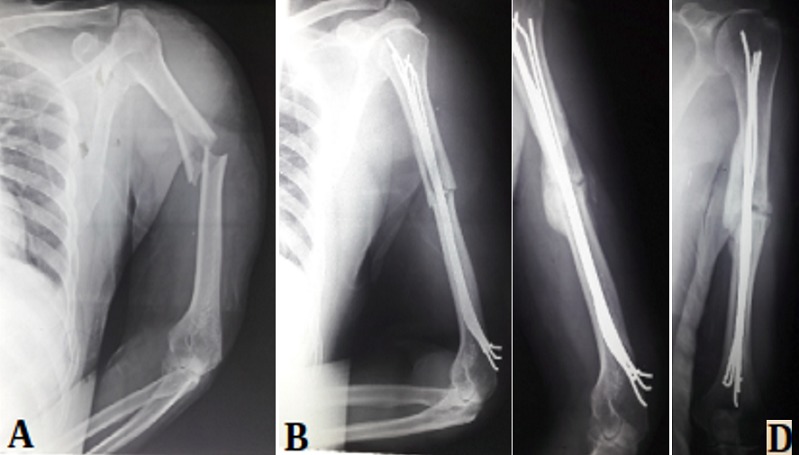
A) patient de 56 ans admis pour polytraumatisme; B) radio à 2 mois post-opératoire; (C, D) radio à 5 mois post-opératoire montrant un retard de consolidation

**Figure 5:**
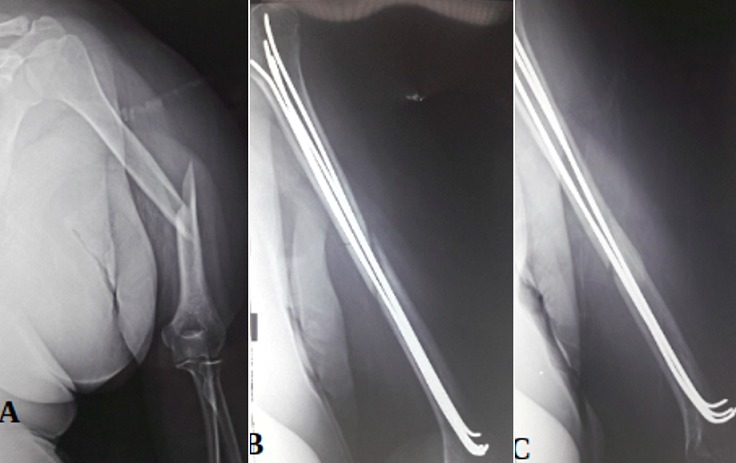
A) jeune femme de 33 ans admise pour fracture spiroïde de la diaphyse humérale; B) radio à j 21 après ablation de l’attelle; C) radio à 2 mois avec consolidation de la fracture

**Figure 6:**
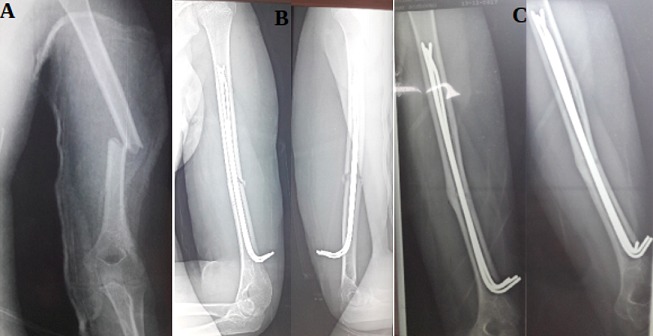
A) jeune homme de 28 ans admis pour fracture transversale de la diaphyse humérale; B) embrochage Hackethal par 3 broches 30/10 avec comblement du canal médullaire (J21)

Il y avait 47 hommes et 4 femmes, L'âge moyen est de 29 ans (12-61). La fracture a intéressé le côté droit chez 19 patients et le côté gauche chez 32 patients (41% du coté dominant). Les circonstances de survenue sont marquées par la fréquence des accidents de la circulation (41 cas), 4 cas d'accidents domestiques, 3 cas d'accidents de travail, 2 cas d'accidents de sport et 1 cas d'agression (Tableau 2). Le siège de la fracture a été déterminé selon la classification d'Hackethal modifiée par De La Caffinière [3] (Tableau 3). Le type de trait de fracture a été précisé selon la classification de l'AO; Les fractures les plus fréquentes étaient de type A3 en zone moyenne D4 (Tableau 4). Dans 27 cas (52,94%) il y avait une ou plusieurs lésions associées dont 5 polytraumatisés, 6 traumatismes étagés du membre supérieur (1 coude flottant) et 7 fractures du membre controlatéral (aucune fracture bilatérale des 2 humérus n´a été retrouvée dans la série). La fracture était ouverte chez 2 patients (stade I selon la classification de Cauchoix et Duparc) et une paralysie radiale sensitivo-motrice post-traumatique a été constatée chez 3 patients (5,88%). Le délai moyen entre le traumatisme et la chirurgie était de 3,4jours. Nous avons trouvé 2 cas (3,92%) de trouble neuroalgodystrophique, mais pas de fracture iatrogène, ni de paralysie radiale post opératoire, 3 cas d'infection au niveau de l'orifice d'entrée des broches mais bien traitées par les soins locaux. Aucune migration de broche à l'épaule ou au coude n'a été relevée. 3 cas (5,88%) de retard de consolidation d´une moyen de 4,5 mois, 4 cas (7,84%) de pseudarthrose aseptique (Figure 4) et 5 cas (9,8 %) de douleur résiduelles du coude en relation avec une tendinite du tendon tricipital bien amélioré sous traitement médical. La consolidation a été obtenue dans 47 cas (92,16%). Le délai moyen de consolidation a été de 10 semaines avec des extrêmes de 7 semaines à 20 semaines (Figure 5, Figure 6). Lors de l'évaluation des résultats fonctionnels, nous avons eu 42 cas (82,35%) de très bons résultats, 4 cas (7,84%) de bons résultats, 1 cas (1,96%) de résultat assez bien et 4 cas (7,84%) de mauvais résultats. Ces derniers étaient des cas de pseudarthrose. Les 3 cas de paralysie radiale post-traumatique ont récupéré, ils ont eu de bons résultats. L´ablation des broches était faite dans les délais de 6 à 12 mois. Sur le plan fonctionnel, l'utilisation du membre supérieur atteint était possible au-dessus de la tête pour 88% des opérés. La mobilité de l'épaule était normale dans 42 cas, déficitaire de 20° en flexion et abduction dans 8 cas et limité dans un cas (déficitaire de 60° en flexion et abduction). La mobilité du coude était normale dans 47 cas, déficitaire de 20° d'extension dans 4 cas. Au dernier recul, 39 patients (76,47%) rapportent qu´ils ont repris leur activité antérieure.

**Tableau 2:** Les circonstances de survenue

circonstances de survenue	%
Accidents de la circulation	80,45
Accident domestique	7,78
Accident de travail	5,88
Accident de sport	3,9
Agression	1,9

**Tableau 3:** Classification du siège de la fracture

Siège de la fracture	Nombre
D2	1
D3	5
D4	40
D5	5

**Tableau 4:** Le type de trait de fracture selon la classification de l’AO

Le type de trait de fracture (AO)	Nombre
A1	4
A2	13
A3	27
B1	2
B2	4
C2	1

## Discussion

Les fractures de la diaphyse humérale posent un problème thérapeutique avec plusieurs complications. Notre taux de 5,88% de paralysie radiale post-traumatique est comparable à la moyenne de la littérature: 8,6% pour Diémé [4], 7,73% pour Putz [5], 10% pour Coudane [6]. Considérant qu´il existe un accord général pour l´exploration chirurgicale des paralysies du nerf radial associée à une fracture ouverte de l´humérus, le traitement approprié des fractures fermées compliqué par paralysie du nerf radial est encore en débat. Ceux en faveur de l´abstention en urgence prétendent que la guérison spontanée se produit dans la majorité des cas. En effet, la récupération spontanée de la fonction du nerf radial après de telles blessures est obtenue dans 73-92% des cas [7]. Holstein A. et al [8] préconisent une exploration systématique face à ces paralysies radiales. Comme De Mourgues [9] l'abstention en urgence et une exploration entre 3 et 4 mois en cas de non récupération a été notre attitude. Les 3 patients ont récupéré leurs fonctions nerveuses. La paralysie radiale secondaire lors de l´ostéosynthèse par plaque est de 6,5 à 12 % [1], ce qui n´est pas négligeable. Pour l´enclouage antérograde, elle est de 4 % Blum et al [10], pour Crates et al [11] et 0 % pour Crolla et al [12] mais le risque de la lésion de la coiffe des rotateurs est important avec limitation de l´abduction de l´épaule avec un taux de 19% de limitations de l'abduction > 20° pour Shvingt et al [13]. L'embrochage centro-médullaire à foyer fermé a l'avantage d'être une technique opératoire simple qui évite les risques inhérents à l'ouverture du foyer de fracture et diminue le risque de lésion du nerf radial et le risque septique [14]. Dans la littérature comme dans notre série, l´embrochage de Hackethal est lié à un faible taux de complication, Le taux de pseudarthrose est comparable au notre et qui est de 7,84%, 2% pour Putz [5], 4,6% pour Gayet [15], 12% pour Zaraa al [16]. Par contre, il est de 27,6% pour André [17]. Même taux de complication retrouver dans la littérature lors de l´ostéosynthèse par plaque et qui varie entre 2,8 à 21 % selon les séries pour la pseudarthrose et 0,8 à 2,4 % pour l´infection [1].

La plupart des problèmes mécaniques rencontrés après embrochage centro-médullaire élastique de Hackethal sont dus aux montages imparfaits aux qualités mécaniques insuffisantes. Ceci se voient essentiellement en cas de broches trop courtes ne prenant pas appui dans le spongieux métaphysaire, de broches de petit calibre ne permettant pas de combler le canal médullaire, de broches dont les courbures initiales sont différentes entraînant une angulation résiduelle ou d'enroulement d'une des broches autour de l'autre empêchant les sommets des courbures de s'écarter et perdant ainsi l'appui cortical [16]. Les principales explications des complications rencontrées lors de notre série sont le défaut d'impaction du foyer, le comblement insuffisant du canal médullaire, le grand nombre de polytraumatisés et de traumatismes à haute énergie. La migration des broches, qui est de 7% pour Gayet [15], causé par le défaut de blocage au niveau de la fenêtre corticale, n´a pas était constatée dans notre série. Il faut noter que la plupart des opérateurs trouvent des difficultés lors de l´introduction des broches d'où l'intérêt de réaliser un trous ovale permettant d´introduire un maximum de broche permettant ainsi de réduire le temps de consolidation et le risque de pseudarthrose même si certains auteurs ont rapporté qu´avec 2 broches on peut assurer un montage stable comme Mc Kibbin [18]. Au cours de notre expérience, on a privilégier l´abord sus-olécranien puisqu´on a remarqué que l´abord épicondylien ne permet pas l´introduction d´un nombre assez élevé de broches pour combler le canal médullaire du faite que le pilier externe est oblique avec une forme évasée de la palette humérale vue de face. Les délais de consolidation sont conformes à ceux de la littérature. 10 semaines pour notre série, 9,4 semaines pour Durbin [19] et 8,5 semaines pour Putz [5]. L´ostéosynthèse par plaque permet d´obtenir la consolidation en 11 à 19 semaines [1]. L´enclouage antérograde permet une consolidation en 12,6 semaines pour Ingman et Water [20] et 13,7 pour Rommens et al [21]. Nous avons rapporté 7,84% de déficit d'extension du coude, ce qui est comparable avec la littérature, 25% pour Putz [5]. Sur le plan biomécanique ont expérimentalement démontré la relative instabilité d'un embrochage huméral qui ne contrôle en fait que partiellement les contraintes rotatoires. Mais les études cliniques ainsi que notre série, montrent que l´embrochage élastique d´Hackethal donne de très bons résultats [14].

## Conclusion

L'embrochage fasciculé selon Hackethal est une solution de compromis entre les méthodes orthopédiques et les ostéosynthèses rigides : l'ostéosynthèse par clous peuvent entraîner des lésions de la coiffe des rotateurs, les Plaques exposent aux risques du foyer ouvert en plus du risque de lésion du nerf radial et le traitement orthopédique apporte moins de confort au patient. Quant au coût, il reste modeste avec une innocuité importante même lors de l'ablation du matériel [12]. Mais il faut souligner que la simplicité technique de l'embrochage n'est qu'apparente. Si les règles sont respectées et l'opérateur a une bonne expérience, l'embrochage devient une technique fiable, rapide et sure.

### Etat des connaissances actuelles sur le sujet

- Traitement chirurgical des fractures diaphysaires de l'humérus fait appelle à plusieurs techniques, comportant des risques de lésion du nerf radial et de la pseudarthrose;
- La simplicité technique de l'embrochage n'est qu'apparente et nécessite une période d'apprentissage.

### Contribution de notre étude à la connaissance

- La voie d'abord sus-olécranienne est mieux adapté pour l'introduction d'un maximum de broches par rapport à la voie d'abord épicondylien;
- L'exploration systématique du nerf radial n'est pas d'actualité, et une surveillance de 2 à 4 mois est souhaitable afin de dépister une éventuelle récupération;
- Le faible taux de complication et la nature mini-invasive sont les avantages de cette méthode.

## Conflits d’intérêts

Les auteurs ne déclarent aucun conflit d'intérêts.
